# Evaluation of the novel ultrasound score for large joints in psoriatic arthritis and ankylosing spondylitis: six month experience in daily clinical practice

**DOI:** 10.1186/1471-2474-14-358

**Published:** 2013-12-19

**Authors:** Valentin S Schäfer, Martin Fleck, Herbert Kellner, Johannes Strunk, Horst Sattler, Wolfgang A Schmidt, Boris Ehrenstein, Marina Backhaus, Wolfgang Hartung

**Affiliations:** 1 Department of Rheumatology / Clinical Immunology, Asklepios Clinic, Kaiser-Karl-V-Allee 3, Bad Abbach, Germany; 2 Department Medical Clinic I, University Regensburg, Franz-Josef-Strauß-Allee 11, Regensburg, Germany; 3 Hospital Neuwittelsbach, Romanstraße 9, Munich, Germany; 4 Hospital Porz am Rhein, Urbacher Weg 19, Cologne, Germany; 5 Medizinische Klinik A, Klinikum Ludwigshafen, Bremserstraße 79, Ludwigshafen, Germany; 6 Medical Center for Rheumatology Berlin-Buch, Lindenberger Weg 19, Berlin, Germany; 7 Charité-University Medicine Berlin, Charitéplatz 1, Berlin, Germany

**Keywords:** Psoriatic arthritis, Ankylosing spondylitis, Large joints, Ultrasound, Scoring

## Abstract

**Background:**

To evaluate the utility of the recently introduced SOLAR score (sonography of large joints in Rheumatology), which has been validated in RA patients, in a cohort of patients with Psoriatic Arthritis (PsA) and Ankylosing Spondylitis (AS) presenting with involvement of large peripheral joints.

**Methods:**

The recently established SOLAR score has been designed to determine the degree of inflammation in the shoulder, the elbow, the hip and the knee joint in patients suffering from RA. Since large joints are frequently involved in PsA and AS, synovitis and synovial vascularity were scored semiquantitatively (grade 0–3) by grey scale (GSUS) and power Doppler ultrasound (PDUS) utilizing the validated scoring system. Each joint was scanned from different angles, the knee joint for example was divided into four areas to score for synovitis: the suprapatellar longitudinal, the medial longitudinal, the lateral longitudinal, and the posterior region. Each area was scored from 0–3, so a maximum score of 12 could be achieved. PsA and AS patients presenting with peripheral joint disease involving large joints were examined at baseline, 3 and 6 months after initiation of local or systemic therapy (DMARDs/Biologics). For evaluation of the inflammatory status, the erythrocyte sedimentation rate (ESR) was determined.

**Results:**

A cohort of 126 patients were enclosed, and 83 of these were followed for 6 months. At baseline before modification of the therapy, patients received DMARDs (n = 83), DMARDs plus biologics (n = 30), or biologic monotherapy (n = 29). Following intervention, all US scores demonstrated a marked improvement. The GSUS and the PDUS scores for all joint areas, except the PDUS score of the hip, exhibited a significant improvement (p < 0.05), while the GSUS of the knee showed even a highly significant (p < 0.001) change. The ESR displayed a significant decrease from 27 to 19 mm (p < 0.002) representing good treatment response.

**Conclusion:**

The SOLAR score, which has been recently introduced for RA patients, is a very suitable instrument for the qualitative and quantitative evaluation of large joint involvement in PsA and AS patients and allows for treatment monitoring.

## Background

Musculoskeletal ultrasound (US) is a valuable imaging tool in rheumatic diseases and has been increasingly used in rheumatologic practice and research in the last decade. Compared with clinical examination, grey scale ultrasound (GSUS) is a more sensitive method for detecting synovitis and tenosynovitis. Therefore, several ultrasound scores, mainly for RA, have been introduced recently to estimate the inflammatory activity and the therapeutic response [1]. So far, mostly small joints such as the MCP-, PIP-, MTP-joints and wrists were enrolled into the scanning protocols, being the most affected joints in RA. However, also large joints are frequently involved in the arthritic process particularly in spondyloarthritis (SpA). Concerning psoriatic arthritis (PsA), the majority of publications have focused on entheseal inflammation, while surprisingly few articles have reported on synovial inflammation or hyperemia, which are characteristic features of large joint involvement in this condition [2]. In ankylosing spondylitis (AS) typically affecting the spine, the most frequent single peripheral manifestation is peripheral arthritis (46.6%), followed by enthesitis (9.8%) and dactylitis (1.9%) [3]. GSUS and power Doppler ultrasound (PDUS) exhibit a higher sensitivity in detecting inflammation of large joints compared with clinical examination [4,5]. Despite this fact, no US score for large joint involvement has been yet developed for PsA and AS. Therefore, the main focus of this project was the establishment of an US score for large joints, as recently published by our group for rheumatoid arthritis (RA) [6], in patients with PsA and AS, suitable for daily rheumatologic practice.

## Methods

A total of 126 patients suffering from PsA or AS and presenting with arthritic manifestation of at least one large joint underwent US examination. All PsA patients had to fulfill the CASPAR criteria [7], while all AS patients had to fulfill the ASAS classification criteria for spondyloarthritis in order to be included [8]. 83 of these patients already concluded the 6 months visit. The clinically dominant joint was evaluated sonographically after the initiation of therapy, or escalation of the established treatment.

Each patient gave his written informed consent for participation of the study.

The study was approved by the ethics committee of the University of Regensburg.

### Clinical assessment

At baseline, and after 3 and 6 months, bilateral hips, elbows, shoulders and knees were clinically assessed for swelling and tenderness. In addition, the following data were recorded on report sheets: year of birth, sex, height, weight, onset of typical symptoms, current rheumatologic therapy including DMARDs, biologics, and nonsteroidal antiinflammatory drugs, as well as systemic and intraarticular glucocorticoid dosage at each visit.

### Laboratory evaluation

Erythrocyte sedimentation rate (ESR, normal levels < 20 mm/hour) was obtained at each visit.

### US examination

As recently published for RA [6], the most affected large joint was sonographically examined in a standardized modified manner according to the German [9] and European League against Rheumatism (EULAR) [10] guidelines at baseline and the follow up visits. All joint regions were assessed by GSUS and PDUS.

### GSUS

Synovitis by GSUS was analyzed semiquantitatively from 0 to 3 (0 = absence, 1 = mild, 2 = moderate, 3 = severe, Table 1). Grade 1 represented a small abnormal hypoechoic/anechoic line beneath the joint capsule. For grade 2, the joint capsule is elevated parallel to the joint area. Grade 3 characterizes a strong convex distension of the joint (Table 1).

**Table 1:** Overview of the sonographic regions scanned

**Region**	**Plane**	**Grade 0**	**Grade 1**	**Grade 2**	**Grade 3**
Shoulder	Dorsal transverse	Normal	Effusion/synovitis in external rotation only	Effusion/synovitis in external and internal rotation	Remarkable convex JCD
Shoulder	Axillary longitudinal	Normal	Joint capsule distension (JCD) concave	JCD straight	JCD convex
Elbow	Humero-radial	Normal	JCD parallel to the humerus	JCD straight	JCD convex
Elbow	Humerulnar	Normal	JCD paralel to the humerus	JCD straight	JCD convex
Elbow	Olecranon fossa	Normal	Olecranon fossa partially filled	Fossa olecrani completely filled	JCD convex above the olecranon fossae
Hip	Anterior longitudinal	Normal	JCD concave	JCD straight	JCD convex
Knee	Suprapatellar longitudinal	Normal	JCD parallel to the humerus	JCD straight	JCD convex
Knee	Medial/lateral longitudinal	Normal	JCD parallel to bone no distension over the joint space	JCD parallel to bone distension above the joint space	JCD convex above the joint space
Knee	Posterior	Normal	Slight JCD over the jointspace	JCD parallel to bone, distension over the joint space	JCD convex above the joint space

### PDUS

PDUS was performed for synovitis and tenosynovitis in each scanning plane. The semiquantitative findings of PDUS activity for synovitis were scored as follows: Grade 0 = no intraarticular color signal, grade 1 = up to 3 color signals representing only low flow, grade 2 = greater than grade 1 to < 50% of the intraarticular area filled with color signals representing clear flow, grade 3 = > 50% of the intraarticular area filled with color signals [1].

Based on these results, a score for each large joint was established, including the sum of the synovitis scores in the GSUS and the PDUS modes. Depending on the number of scored planes, the score values are different for the shoulder (GSUS/PDUS 0–6), the elbow (GSUS/PDUS 0–9), the hip (GSUS/PDUS 0–3) and the knee (GSUS 0–12 and PDUS 0–15). The maximum GSUS score of 12 for the knee is explained by scanning four areas of the knee, the suprapatellar longitudinal, the medial longitudinal, the lateral longitudinal, and the posterior region, each assigning a grade from 0 to 3. In PDUS we scored the same areas of the knee adding an additional infrapatellar longitudinal scan and grading it from 0 to 3, yielding a total possible score of 15.

### US inter- and intrareader reliability

The results regarding inter- and intrareader reliability testing for the SOLAR-score have been recently published [6].

### Statistical analysis

Statistical analysis was performed with SPSS statistical software, version 17.02 (SPSS, Chicago, IL). For quantitative parameters (e.g., number of patients, age of examined patients, and their disease activity), the mean and +/− SD and range were determined. Significant changes were calculated by the Wilcoxon Test. P-values less than 0.05 were considered statistically significant.

## Results

### Characteristics of patients

Eighty three patients (51.8% women) with a mean age of 45.3 ± 14.5 years were examined at three visits (baseline, and following 3 and 6 months). 59% of patients suffered from PsA and 41% from AS with peripheral joint involvement present in all patients.

### Medication

At start of the observation, a total of 29% of the patients received steroids (42% systemic administration, 6% systemic and local intraarticular administration and 6% local intraarticular injections into the target joint only).Patients were treated with either DMARDs (49.4%) a combination of DMARDs plus biologics (14.7%) or biologic monotherapy (28.9%). A possible change in medication over the six months was not recorded.

### US and laboratory parameters

US, and laboratory results are depicted in Table 2 for the entire group.

**Table 2:** Ultrasound and laboratory results for the entire patient group

	**US and laboratory data (n = 83)**^**#**^			
**Joint region**	**US score/disease activity**	**Baseline**	**After 3 months**	**After 6 months**
Shoulder (n = 11)	GSUS (range 0–6)	2,8 ± 1.9	1,9 ± 1.5	1.1 ± 1.2*
	PDUS (range 0–6)	1.7 ± 1.6	0,7 ± 1.0	0.4 ± 0.8*
Elbow (n = 10)	GSUS (range 0–9)	4,3 ± 2.6	2.1 ± 1.9	0.9 ± 1.5*
	PDUS (range 0–9)	2.3 ± 2.0	1.0 ± 1.2	0.6 ± 1.1*
Hip (n = 13)	GSUS (range 0–3)	2.0 ± 0.8	1.3 ± 0.8	1.0 ± 1.1*
	PDUS (range 0–3)	0.7 ± 0.9	0.3 ± 0.5	0.3 ± 0.5
Knee (n = 49)	GSUS (range 0–12)	5.3 ± 2.9	3.5 ± 2.7	2.8 ± 2.8**
	PDUS (range 0–15)	2.9 ± 3.1	1.5 ± 1.9	1.4 ± 2.4*
ESR, mm/hour		27.3 ± 22.6	18.0 ± 14.7	18.8 ± 16.1*

^#^values represent the mean ± SD. US = ultrasound; GSUS = gray scale US; PDUS = power doppler US; ESR = erythrocyte sedimentation rate. *p < 0.05. **p < 0.001.

## Discussion

PsA and peripheral AS are sometimes considered as benign forms of arthritis, however, these disease manifestations affect patient’s quality of life substantially, and also cause significant functional impairment [11].

Therefore, and considering the low sensitivity of clinical assessment concerning inflammatory changes in large joints, [5] the recently published US score for large joints in RA patients has been applied in a cohort of PSA and AS patients [12].

Two standardized scanning planes were defined for the shoulder joint (the posterior transverse scan and the axillary longitudinal scan). According to published data and our own experience, these scanning planes allow for detection of up to 95% of effusions/synovitis in the glenohumeral joint [13]. Furthermore, these two planes are easy to perform and fast to apply.

The scanning protocol for the elbow covers the anterior humeroradial, the anterior humeroulnar and the posterior longitudinal scan over the olecranon fossa facilitating the delineation of the joint capsule and pathologic distensions. These planes have been successfully utilized for detection of effusion and synovitis in the elbow joint [14].

The anterior longitudinal scan was chosen for the hip to be the best for scoring of synovitis and hypervascularisation as previously proposed by Boutry et al. [15]. In addition, a high correlation between histopathologically confirmed vascularity and PDUS signal activity has been demonstrated for the anterior longitudinal scan [16].

The knee was scanned in GSUS in four standardized planes - the suprapatellar longitudinal, the medial longitudinal, the lateral longitudinal, and the dorsal plane. The infrapatellar longitudinal scan was additionally added for the PDUS score, whereas only the suprapatellar longitudinal scan and the medial and lateral recessus have been analyzed in previous studies to estimate the inflammatory activity.

Previously, the synovial thickness and power Doppler flow have been measured in the suprapatellar and parapatellar pouch, whereas the knee has been scanned in the suprapatellar and parapatellar planes by other investigators [17]. According to our experience, the suprapatellar and especially the medial and lateral longitudinal scans are most suitable to detect hypervascularisation of an inflamed knee joint, hence we chose these planes for our protocol.

Utilizing the novel SOLAR score [12] for longitudinal follow up of our PsA and AS cohort, a highly significant reduction (p < 0.001) has been observed in GSUS scores for the knee within 6 months of follow up. In addition, PDUS and GSUS scores for for all joint areas improved significantly (p < 0.05), with the only exception being the PDUS of the hip. The decrease of systemic inflammation as demonstrated by ESR reduction (p < 0.002) are reflected in the SOLAR scores supporting the potential of this scoring system to assess disease activity and monitor treatment response of large joint involvement in PsA- and AS patients.

A major limitation of the study is the lack of a “gold standard” to adjust the ultrasound scoring. However, to our knowledge, there have been no MRI scores developed and published for arthritis in large joints, and conventional x-ray scoring systems do not address soft tissue alterations. Therefore, no other imaging modality could be applied to validate the results of the SOLAR score obtained in PsA- and AS patients.

## Conclusion

In conclusion, the SOLAR score is a valuable tool for the US examination of inflamed large joints in patients with PSA and AS. If large joints are affected, the SOLAR score is a time-effective monitoring tool in daily rheumatological practice.

## Competing interests

All authors declare that they have no financial or non-financial competing interests.

## Authors’ contributions

WH made substantial contributions to conception and design and acquisition of data, and have been involved in drafting the manuscript and revising it critically for important intellectual content; moreover he give final approval of the version to be published. VSS has been drafting the manuscript and revising it critically for important intellectual content. MB, HS, HK and JS made substantial contributions to the Study design and acquisition of data. BE and MF contributed to the analysis and interpretation of data and have been involved in revising the manuscript critically for important intellectual content and have given final approval of the version to be published. All authors read and approved the final manuscript.

## Pre-publication history

The pre-publication history for this paper can be accessed here:

http://www.biomedcentral.com/1471-2474/14/358/prepub

## References

[B1] Backhaus M Ohrndorf S Kellner H Strunk J Backhaus TM Hartung W Evaluation of a novel 7-joint ultrasound score in daily rheumatologic practice: a pilot project Arthritis Rheum 2009 14 9 1194 1201 10.1002/art.24646 19714611

[B2] Dhir V Aggarwal A Psoriatic Arthritis: a Critical Review Clin Rev Allergy Immunol 2013 14 2 141 148 10.1007/s12016-012-8302-6 22294201

[B3] Rudwaleit M van der Heijde D Landewe R Akkoc N Brandt J Chou CT The Assessment of SpondyloArthritis International Society classification criteria for peripheral spondyloarthritis and for spondyloarthritis in general Ann Rheum Dis 2011 14 1 25 31 10.1136/ard.2010.133645 21109520

[B4] Luukkainen R Sanila MT Luukkainen P Poor relationship between joint swelling detected on physical examination and effusion diagnosed by ultrasonography in glenohumeral joints in patients with rheumatoid arthritis Clin Rheumatol 2007 14 6 865 867 10.1007/s10067-006-0402-3 17031485

[B5] Naredo E Aguado P De Miguel E Uson J Mayordomo L Gijon-Banos J Painful shoulder: comparison of physical examination and ultrasonographic findings Ann Rheum Dis 2002 14 2 132 136 10.1136/ard.61.2.132 11796399 PMC1754006

[B6] Hartung W Kellner H Strunk J Sattler H Schmidt WA Ehrenstein B Development and evaluation of a novel ultrasound score for large joints in rheumatoid arthritis: one year experience in daily clinical practice Arthritis Care Res 2012 14 5 675 682 10.1002/acr.21574 22183834

[B7] Taylor W Gladman D Helliwell P Marchesoni A Mease P Mielants H Classification criteria for psoriatic arthritis: development of new criteria from a large international study Arthritis Rheum 2006 14 8 2665 2673 10.1002/art.21972 16871531

[B8] Sieper J Rudwaleit M Baraliakos X The Assessment of SpondyloArthritis international Society (ASAS) handbook: a guide to assess spondyloarthritis Ann Rheum Dis 2009 14 Suppl 2 ii1 ii44 19433414 10.1136/ard.2008.104018

[B9] Schmidt WA Hauer RW Banzer D Bohl-Buhler M Braun J Mellerowicz H Technique and value of arthrosonography in rheumatologic diagnosis. 2: ultrasound diagnosis of the hip area Z Rheumatol 2002 14 2 180 188 10.1007/s003930200028 12056297

[B10] Backhaus M Burmester GR Gerber T Grassi W Machold KP Swen WA Guidelines for musculoskeletal ultrasound in rheumatology Ann Rheum Dis 2001 14 7 641 649 10.1136/ard.60.7.641 11406516 PMC1753749

[B11] Paramarta JE Baeten D De Rycke L Synovial Tissue Response to Treatment with TNF Blockers in Peripheral Spondyloarthritis Open Rheumatol J 2011 14 127 132 10.2174/1874312901105010127 22279512 PMC3263444

[B12] Hartung W Kellner H Strunk J Sattler H Schmidt WA Ehrenstein B Development and evaluation of a novel ultrasound score for large joints in rheumatoid arthritis: one year experience in daily clinical practice Arthritis Care Res 2011 14 5 675 682 10.1002/acr.21574 22183834

[B13] Schmidt WA Schicke B Krause A Which ultrasound scan is the best to detect glenohumeral joint effusions? Ultraschall Med 2008 14 Suppl 5 250 255 18484062 10.1055/s-2008-1027330

[B14] Koski JM Ultrasonography of the elbow joint Rheumatol Int 1990 14 3 91 94 10.1007/BF02274820 2203136

[B15] Boutry N Khalil C Jaspart M Marie-Helene V Demondion X Cotten A Imaging of the hip in patients with rheumatic disorders Eur J Radiol 2007 14 1 49 58 10.1016/j.ejrad.2007.03.021 17543486

[B16] Walther M Harms H Krenn V Radke S Kirschner S Gohlke F Synovial tissue of the hip at power Doppler US: correlation between vascularity and power Doppler US signal Radiology 2002 14 1 225 231 10.1148/radiol.2251011272 12355009

[B17] Carotti M Salaffi F Manganelli P Salera D Simonetti B Grassi W Power Doppler sonography in the assessment of synovial tissue of the knee joint in rheumatoid arthritis: a preliminary experience Ann Rheum Dis 2002 14 10 877 882 10.1136/ard.61.10.877 12228155 PMC1753902

